# Epidemiologic Study of Intensive Care Unit Admission in South Korea: A Nationwide Population-Based Cohort Study from 2010 to 2019

**DOI:** 10.3390/ijerph20010081

**Published:** 2022-12-21

**Authors:** Tak-Kyu Oh, Hyeong-Geun Kim, In-Ae Song

**Affiliations:** 1 Department of Anesthesiology and Pain Medicine, Seoul National University Bundang Hospital, Seongnam 13620, Republic of Korea; 2 Department of Anesthesiology and Pain Medicine, College of Medicine, Seoul National University, Seoul 04551, Republic of Korea

**Keywords:** cohort studies, critical care, epidemiology, intensive care units, mortality

## Abstract

We aimed to investigate the trends of intensive care unit (ICU) admissions in South Korea from 2010 to 2019. We included all adult patients (≥20 years old) who were admitted to the ICU during hospitalization from 2010 to 2019 in South Korea. There were 3,517,423 ICU admissions of 2,461,848 adult patients. Of the ICU admission cases, 66.8% (2,347,976/3,517,423) were surgery-associated admissions, and the rate of in-hospital mortality after ICU admission was 12.0% (422,155 patients). The most common diagnoses were diseases of the circulatory system (36.8%) and pneumonia (4%). The 30-day, 90-day, and 1-year mortality rates were 16.0%, 23.6%, and 33.3% in 2010, and these values slightly decreased by 2019 to 14.7%, 22.1%, and 31.7%, respectively. The proportions of continuous renal replacement therapy (CRRT) use and extracorporeal membrane oxygenation (ECMO) support were 2.0% and 0.3% in 2010, and these values gradually increased by 2019 to 4.7% and 0.8%, respectively. Although the age and cost of hospitalization among critically ill patients who were admitted to the ICU increased from 2010 to 2019, the mortality rate decreased slightly. Moreover, the proportions of ECMO support and CRRT use had increased in our South Korean cohort.

## 1. Introduction

The intensive care unit (ICU) is designed to care for critically ill patients who require more support and attention than is available in the general ward [1]. The first intensive care unit (ICU) was established in the late 1950s, and since then, critical care medicine has improved [2,3]. Currently, the ICU plays a critical role in monitoring critically ill patients and providing interventions and organ support [4].

Although critical care medicine and ICUs have a 60-year-long history [5], there are not enough epidemiologic studies on the trends of ICU admission based on big data. Most epidemiological studies have analyzed the trends of surgical or neurological ICU admis-sions [6,7]. Garland et al. reported ICU admission trends using population-based data of all adult ICUs in the Canadian province of Manitoba from 1999 to 2007 [8]. Weissman et al. reported the ICU admission trends of Medicare beneficiaries from 2006 to 2015 in the United States [9]. However, the circumstances of ICU admission differ among countries, depending on the availability of resources for organ support and adequate staffing [10]. In South Korea, the National Health Insurance Service (NHIS) provides nationwide registra-tion data for medical research, including treatment information related to ICU admissions. Thus, using data from the NHIS database, we examined the trends of ICU admission in South Korea from 2010 to 2019. This time frame (2010–2019) was chosen because many advances have been made in critical care medicine in South Korea since 2010 [11].

## 2. Materials and Methods

### 2.1. Ethical Statement

This population-based cohort study complied with the Strengthening the Reporting of Observational Studies in Epidemiology guidelines [12]. The study protocol was approved by the Institutional Review Board (X-2102-666-904) and the Big Data Center of the NHIS (NHIS-2021-1-620). The requirement for informed consent was waived because the data analyses were performed retrospectively using anonymized data derived from the South Korean NHIS database.

### 2.2. Data Source and Study Population

As a single public health insurance system, the NHIS contains and manages the data on disease diagnoses and prescriptions of procedures and/or drugs. In South Korea, physicians (from all outpatient clinics and hospitals) must register all prescription information on procedures, medications, and disease diagnoses in the NHIS database to receive treatment costs from the government. Diseases are registered using the International Classification of Diseases External 10th Revision (ICD-10 codes). Moreover, the NHIS database contains demographic and socio-economic status-related information of all the patients in South Korea.

We included all adult patients (≥20 years) who were admitted to the ICU during hospitalization from 2010 to 2019 in South Korea. The prescription code of ICU admission during hospitalization, available in the NHIS, was used for data extraction.

### 2.3. Data Collection

The following demographic and socioeconomic data were collected: age, sex, em-ployment status, national household income level, and residence at hospital admission. The NHIS contains the patients’ household income level that is used to determine insurance premiums in the year, and approximately 67% of the medical expenses are subsidized by the government [13]. However, individuals from low-income households are enrolled in the Medical Aid program; in this program, the government covers nearly all medical expenses to minimize the financial burden of medical costs. The patients were divided into five groups using quartile ratios (Q1 to Q4 groups and Medical Aid program group). Residence was classified into urban (Seoul and other metropolitan cities) and rural residence (all other areas). The lengths of hospital stay (days) and ICU stay were recorded. The admitting departments were classified into internal medicine [IM] and non-IM. We also reported whether the patients were admitted to the ICU through the emergency room (ER). The patients who underwent surgery during hospitalization were considered to have surgery-associated hospital admissions. The hospitals in which the patients were admitted were classified into three groups: tertiary general hospitals, general hospitals, and other hospitals. To determine the comorbid status of the patients, the Charlson comorbidity index (CCI) was calculated using the ICD-10 codes (Table S1). Data on the use of mechanical ventilatory support, extracorporeal membrane oxygenation (ECMO) sup-port, and continuous renal replacement therapy (CRRT) during ICU stay were collected. The follow-up events were classified into four groups: (1) same-hospital follow-up, (2) transfer to a long-term facility care center, (3) death during hospitalization, and (4) discharge and other outpatient clinic follow-up. The dates of death during hospitalization and hospital discharge were also collected. The total cost of hospitalization was collected (in United States Dollar, USD). The main diagnosis at ICU admission was identified using ICD-10 codes. The main diagnosis of all the patients was determined by the NHIS after hospital discharge or death as the disease that required intensive treatment or examination during hospitalization.

### 2.4. Study Objectives

First, the trends of age, total cost, mortality (30-day, 90-day, and 1-year), ECMO support, and CRRT use were examined from 2010 to 2019. Second, we investigated the factors associated with in-hospital mortality and 1-year mortality among all patients admitted to the ICU.

### 2.5. Statistical Analyses

The clinicopathological characteristics of the patients are presented as mean values with standard deviations (SDs) for continuous variables and as numbers with percentages for categorical variables. Multivariable logistic regression modeling was used to determine which factors were associated with in-hospital mortality among patients admitted to the ICU. All covariates were included in the multivariable model for adjustment, and the results were presented as adjusted odds ratios (aORs) with 95% confidence intervals (CIs). The Hosmer–Lemeshow statistic test was used to confirm the goodness of fit of the model. Moreover, we also fitted a multivariable Cox regression model for 1-year mortality among patients admitted to the ICU. The results were presented as adjusted hazard ratios (aHRs) with 95% CIs, and log/log plots were used to confirm that the central assumptions of Cox proportional hazard models were satisfied. There was no multicollinearity between the variables with the criterion of variance inflation factors < 2.0. All statistical analyses were performed using IBM SPSS Statistics for Windows (version 25.0; IBM Corp., Armonk, NY, USA), and statistical significance was set at *p* < 0.05.

## 3. Results

### 3.1. Study Population

From 1 January 2010, to 31 December 2019 (10 years), there were 3,517,423 ICU admissions of 2,461,848 adult patients. The clinicopathological characteristics of the patients are presented in Table 1. The mean age of the patients admitted to the ICU was 67.6 years (SD, 15.3 years), and the proportion of male patients was 56.5% (1,988,579/3,517,423). The mean values of length of ICU and hospital stays were 4.1 (6.0 days) and 15.3 days (13.3 days), respectively. Further, 66.8% (2,347,976/3,517,423) of the ICU admissions were surgery-associated admissions. In-hospital mortality after ICU admission occurred in 422,155 (12.0%) patients. The mean cost of hospitalization per patient was 8049.7 USD (SD, 9043.8 USD).

### 3.2. Diagnosis at ICU Admission

Table 2 lists the main diagnoses at ICU admission. The most common diagnoses were diseases of the circulatory system (I00-I99, 36.8%), followed by neoplasm (C00-D49, 14.1%); injury, poisoning, and certain other consequences of external causes (S00-T88, 10.8%); and diseases of the respiratory system (J00-J99, 10.6%). Table S2 lists the 18 common specific diseases in the main diagnoses at ICU admission. The most common diagnosis was pneumonia (J189, 4%), followed by cerebral infarction (I639, 2.8%), unstable angina (I200, 2.8%), traumatic subdural hemorrhage (S065, 2.4%), acute myocardial infarction (I219, 2.3%), and sepsis (A419, 1.9%).

### 3.3. Trends of ICU Admission

**Figure 1:**
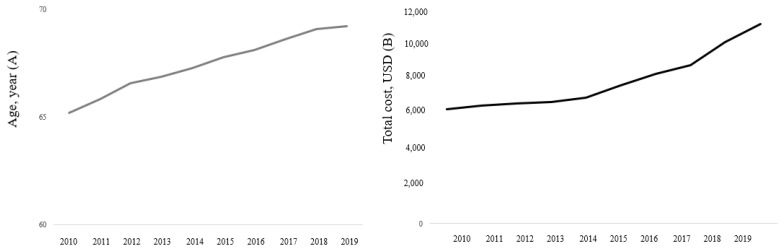
Trends of age (**A**) and total cost (**B**) of hospitalization among patients admitted to the ICU from 2010 to 2019. ICU, intensive care unit.

**Figure 2:**
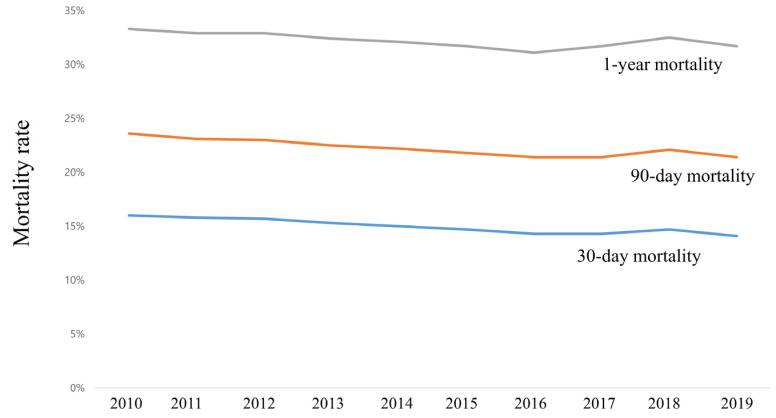
Trends of mortality after ICU admission among patients admitted to the ICU from 2010 to 2019. ICU, intensive care unit.

**Figure 3:**
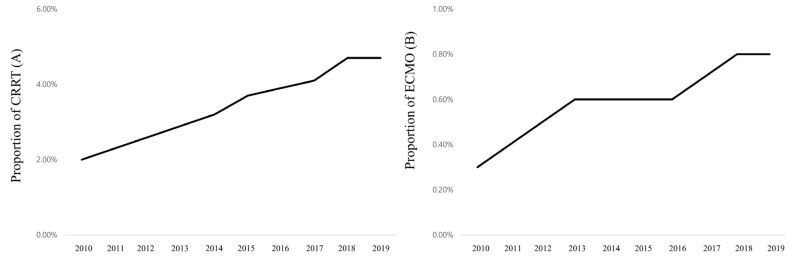
Trends of use of CRRT (**A**) and ECMO (**B**) among patients admitted to the ICU from 2010 to 2019. ECMO, extracorporeal membrane oxygenation; CRRT, continuous renal replacement therapy.

The mean values of age and total cost were 65.2 Â years (SD, 15.4 years) and 6370.6 USD (SD, 6729.0 USD) in 2010, and these values gradually increased to 69.2 years (SD, 15.2 years) and 11,131.5 USD (12,133.3 USD) in 2019 (Figure 1A,B). The 30-day, 90-day, and 1-year mortality rates were 16.0%, 23.6%, and 33.3% in 2010 and slightly decreased in 2019 to 14.7%, 22.1%, and 31.7%, respectively (Figure 2). The proportions of CRRT use and ECMO support were 2.0% and 0.3% in 2010 and gradually increased in 2019 by 4.7% and 0.8%, respectively (Figure 3A,B).

### 3.4. In-Hospital Mortality and 1-Year Mortality

Table 3 shows the results of the multivariate logistic regression model for in-hospital mortality among patients admitted to the ICU in South Korea from 2010 to 2019. Older age (aOR: 1.03, 95% CI: 1.03–1.03; *p* < 0.001), male sex (aOR: 1.08, 95% CI: 1.07–1.08; *p* < 0.001), increased CCI (aOR: 1.08, 95% CI: 1.08–1.09; *p* < 0.001), admission from IM (aOR: 1.82, 95% CI: 1.80–1.83; *p* < 0.001), admission through ER (aOR: 1.39, 95% CI: 1.37–1.40; *p* < 0.001), mechanical ventilatory support (aOR: 8.41, 95% CI: 8.34–8.48; *p* < 0.001), ECMO support (aOR: 2.86, 95% CI: 2.77–2.95; *p* < 0.001), and CRRT use (aOR: 4.38, 95% CI: 4.32–4.44; *p* < 0.001) were associated with increased in-hospital mortality. However, compared with the Medical Aid program group, the Q1 (aOR: 0.89, 95% CI: 0.87–0.90; *p* < 0.001), Q2 (aOR: 0.90, 95% CI: 0.89–0.91; *p* < 0.001), Q3 (aOR: 0.86, 95% CI: 0.85–0.87; *p* < 0.001), and Q4 groups (aOR: 0.83, 95% CI: 0.82–0.84; *p* < 0.001) of household income showed decreased in-hospital mortality. Moreover, patients who were employed at ICU admission showed lower in-hospital mortality than those who were unemployed (aOR: 0.95, 95% CI: 0.94–0.96; *p* < 0.001). Table 4 also shows the results of the multivariate Cox regression model for 1-year mortality among patients admitted to the ICU in South Korea from 2010 to 2019.

## 4. Discussion

In this population-based cohort study, we determined the trends of ICU admission in South Korea from 2010 to 2019. The proportion of surgery-associated ICU admissions was 66.8%, with diseases of the circulatory system being the most common main diagnoses. Among specific diseases, pneumonia was the most common cause of ICU admission. The mean age of the patients admitted to the ICU had increased over the years. In addition, although the total hospital cost at ICU admission increased, the 30-day, 90-day, and 1-year mortality rates had decreased slightly from 2010 to 2019. The proportion of patients who received CRRT or ECMO had also increased. Our results are different from those of previous studies [6,7,8,9] because our survival analysis included all the patients admitted to the ICU. 

We examined the data from 2010 to 2019 to determine the trends of ICU admission in South Korea in this study. In South Korea, on 12 January 2010, a public hearing was held at the National Assembly, where the Korean Society of Critical Care Medicine (KSCCM) expressed the need for intensivists in ICUs to opinion leaders in the legislation, major me-dia outlets, and policy makers [11]. Then, a training system for qualified intensivists was established in South Korea, and there have been advances in this system in the last 10 years. Therefore, it was important to determine the trends of intensive care and hospitali-zation during this period in South Korea.

Interestingly, unemployment and low household income level at ICU admission were associated with a high risk of in-hospital mortality after ICU admission. In the Unit-ed States, low household income was associated with a high risk of in-hospital mortality among patients with sepsis [14]. In our study, in-hospital mortality after ICU admission was high in the Medical Aid program group. Considering that employment reflects the functional status of patients [15], it was not unusual for us to find that unemployment was associated with poor survival outcomes after ICU admission.

Diseases of the circulatory system were the most common diagnoses at ICU admis-sion. The role of the cardiovascular ICU, which is needed for special systemic manage-ment of patients with severe cardiovascular diseases, has recently been emphasized [16]. Moreover, critical care after ICU admission for cardiac surgery has been highlighted as an important factor in recent critical care literature [17]. 

Regarding specific diseases, pneumonia was the most common disease at ICU ad-mission. Both community-acquired and nosocomial pneumonia are common diseases in ICU-admitted patients that may affect the in-hospital mortality of critically ill patients [18,19]. Considering that we focused on data from patients admitted to the ICU until 2019, the prevalence of pneumonia in the South Korean ICU might have increased due to the coronavirus disease pandemic [20].

We found that the proportion of patients receiving both CRRT and ECMO increased from 2010 to 2019. Other epidemiological studies have also reported that the prevalence of CRRT or ECMO has increased in South Korea [21,22]. Moreover, the increase in CRRT or ECMO might increase the total cost of hospitalization at ICU admission, considering that CRRT and ECMO are relatively expensive procedures. This finding is important because the aging population is susceptible to multiorgan dysfunction, eventually requiring CRRT or ECMO, and the use of CRRT or ECMO will continue to increase in the future given the increase in the aging population.

This study had several limitations. First, some important data, such as body mass index or alcohol consumption history, were not included in this study because of the lack of information in the NHIS database. Second, we did not adjust for disease severity in patients admitted to the ICU with a critical illness, namely the acute physiology and chronic health evaluation II score or simplified acute physiology score II were not used for adjustment. Lastly, the generalizability of the results of this study might be limited because the ICU environment and policies of critical care in other countries are different from those in South Korea.

## 5. Conclusions

This study showed that although the age of and cost of hospitalization for critically ill patients who were admitted to the ICU increased from 2010 to 2019, the mortality rate decreased slightly. Diseases of the circulatory system (I00-I99) were the most common main diagnoses, and pneumonia was the most common specific disease at ICU admission. Moreover, the proportions of the use of ECMO and CRRT had increased in South Korea. This study on the recent trends of ICU treatment can help to predict future changes in critical care medicine in South Korea. In addition, our study provides an insight into the aspects of ICU care that need further research and improvement in order to reduce ICU-related mortality in the Korean patient population.

## Figures and Tables

**Table 1:** Clinicopathological characteristics of the patients.

Variable	Mean (SD) or Number (%)
Age, year	67.6 (15.3)
Sex, male	1,988,579 (56.5)
Having a job	1,578,574 (44.9)
Household income level	
Medical Aid program	338,733 (9.6)
Q1 (Lowest)	515,273 (14.6)
Q2	477,117 (13.6)
Q3	612,533 (17.4)
Q4 (Highest)	948,811 (27.0)
Unknown	624,956 (17.8)
Residence	
Urban area	1,181,609 (33.6)
Rural area	1,768,300 (50.3)
Unknown	567,514 (16.1)
ICU stay, day	4.1 (6.0)
LOS, day	15.3 (13.3)
CCI, point	2.4 (2.1)
Admitting department	
Non-IM	1,683,238 (47.9)
IM	1,834,184 (52.1)
Hospital admission through ER	2,035,333 (57.9)
Type of hospital	
Tertiary general hospital	1,393,048 (39.6)
General hospital	2,037,525 (57.9)
Other hospital	86,850 (2.5)
Surgery associated hospital admission	2,347,976 (66.8)
Mechanical ventilator support	928,972 (26.4)
ECMO support	20,710 (0.6)
CRRT use	122,085 (3.5)
Result of hospitalization	
Same-hospital follow-up	876,756 (24.9)
Transfer to a long-term facility care center	157,019 (4.5)
Death during hospitalization	422,155 (12.0)
Discharge and other outpatient clinic follow-up	2,061,493 (58.6)
30 d mortality	525,966 (15.0)
90 d mortality	781,406 (22.2)
1-year mortality	1,132,612 (32.2)
Total cost for hospitalization, USD	8049.7 (9043.8)

SD, standard deviation; LOS, length of hospital stays; CCI, Charlson comorbidity index; IM, internal medicine; ER, emergency room; ECMO, extracorporeal membrane oxygenation; CRRT, continuous renal replacement therapy; USD, United states dollars.

**Table 2:** Main diagnosis at ICU admission.

Main Diagnosis	Number (%)
Certain infectious and parasitic diseases	132,276 (3.8)
Neoplasms	494,762 (14.1)
Diseases of the blood and blood-forming organs and certain disorders involving the immune mechanism	9280 (0.3)
Endocrine, nutritional and metabolic diseases	62,870 (1.8)
Mental, Behavioral and Neurodevelopmental disorders	19,131 (0.5)
Diseases of the nervous system	96,928 (2.8)
Diseases of the eye and adnexa	1571 (0.0)
Diseases of the circulatory system	1,294,520 (36.8)
Diseases of the respiratory system	374,242 (10.6)
Diseases of the digestive system	298,151 (8.5)
Diseases of the skin and subcutaneous tissue	7799 (0.2)
Diseases of the musculoskeletal system and connective tissue	79,724 (2.3)
Diseases of the genitourinary system	159,495 (4.5)
Certain conditions originating in the perinatal period	5948 (0.2)
Congenital malformations, deformations and chromosomal abnormalities	14,415 (0.4)
Symptoms, signs and abnormal clinical and laboratory findings, not elsewhere classified	59,023 (1.7)
Injury, poisoning and certain other consequences of external causes	381,053 (10.8)
Codes for special purposes	493 (0.0)
External causes of morbidity, Factors influencing health status and contact with health services	24,347 (0.7)
Unknown	1395 (0.0)

ICU, intensive care unit.

**Table 3:** Multivariable logistic regression model for in-hospital mortality among patients admitted to the ICU in South Korea from 2010 to 2019.

Variable	aOR (95% CI)	*p*-Value
Age, year	1.03 (1.03, 1.03)	<0.001
Sex, male (vs. female)	1.08 (1.07, 1.08)	<0.001
Having a job (vs. unemployment)	0.95 (0.94, 0.96)	<0.001
Household income level		
Q1 (Lowest) (vs. medical aid program)	0.89 (0.87, 0.90)	<0.001
Q2 (vs. medical aid program)	0.90 (0.89, 0.91)	<0.001
Q3 (vs. medical aid program)	0.86 (0.85, 0.87)	<0.001
Q4 (Highest) (vs. medical aid program)	0.83 (0.82, 0.84)	<0.001
Unknown (vs. medical aid program)	0.86 (0.83, 0.88)	<0.001
Residence		
Rural area (vs. Urban area)	1.00 (0.99, 1.01)	0.506
Unknown (vs. Urban area)	0.73 (0.71, 0.76)	<0.001
CCI, point	1.08 (1.08, 1.09)	<0.001
Admitting department		
IM (vs. non-IM)	1.82 (1.80, 1.83)	<0.001
Hospital admission through ER	1.39 (1.37, 1.40)	<0.001
Type of hospital		
General hospital (vs. tertiary general hospital)	1.73 (1.72, 1.75)	<0.001
Other hospital (vs. tertiary general hospital)	3.94 (3.86, 4.02)	<0.001
Surgery associated hospital admission	0.76 (0.76, 0.77)	<0.001
Mechanical ventilator support	8.41 (8.34, 8.48)	<0.001
ECMO support	2.86 (2.77, 2.95)	<0.001
CRRT use	4.38 (4.32, 4.44)	<0.001
Year of admission		
2011 (vs. 2010)	1.06 (1.04, 1.08)	<0.001
2012 (vs. 2010)	1.04 (1.02, 1.06)	<0.001
2013 (vs. 2010)	1.08 (1.06, 1.10)	<0.00
2014 (vs. 2010)	1.04 (1.02, 1.06)	<0.001
2015 (vs. 2010)	0.99 (0.97, 1.01)	0.285
2016 (vs. 2010)	0.97 (0.96, 0.99)	0.001
2017 (vs. 2010)	0.93 (0.91, 0.95)	<0.001
2018 (vs. 2010)	0.93 (0.91, 0.94)	<0.001
2019 (vs. 2010)	0.92 (0.90, 0.93)	<0.001
Main diagnosis		
Certain infectious and parasitic diseases	1	
Neoplasms	0.87 (0.86, 0.89)	<0.001
Diseases of the blood and blood-forming organs and certain disorders involving the immune mechanism	0.91 (0.86, 0.96)	0.001
Endocrine, nutritional and metabolic diseases	0.32 (0.31, 0.33)	<0.001
Mental, Behavioral and Neurodevelopmental disorders	0.12 (0.11, 0.13)	<0.001
Diseases of the nervous system	0.32 (0.31, 0.33)	<0.001
Diseases of the eye and adnexa	0.12 (0.08, 0.18)	<0.001
Diseases of the circulatory system	0.36 (0.36, 0.37)	<0.001
Diseases of the respiratory system	0.50 (0.49, 0.51)	<0.001
Diseases of the digestive system	0.49 (0.48, 0.50)	<0.001
Diseases of the skin and subcutaneous tissue	0.65 (0.60, 0.71)	<0.001
Diseases of the musculoskeletal system and connective tissue	0.29 (0.28, 0.30)	<0.001
Diseases of the genitourinary system	0.37 (0.36, 0.38)	<0.001
Certain conditions originating in the perinatal period	0.33 (0.28, 0.40)	<0.001
Congenital malformations, deformations and chromosomal abnormalities	0.16 (0.15, 0.18)	<0.001
Symptoms, signs and abnormal clinical and laboratory findings, not elsewhere classified	0.64 (0.62, 0.66)	<0.001
Injury, poisoning and certain other consequences of external causes	0.41 (0.40, 0.42)	<0.001
Codes for special purposes	0.55 (0.42, 0.73)	<0.001
External causes of morbidity, Factors influencing health status and contact with health services	0.36 (0.36, 0.37)	<0.001

ICU, intensive care unit; aOR, adjusted odds ratio; CI, confidence interval; CCI, Charlson comorbidity index; IM, internal medicine; ER, emergency room; ECMO, extracorporeal membrane oxygenation; CRRT, continuous renal replacement therapy.

**Table 4:** Multivariable Cox regression model for 1-year mortality among patients admitted to the ICU in South Korea from 2010 to 2019.

Variable	aHR (95% CI)	*p*-Value
Age, year	1.04 (1.04, 1.04)	<0.001
Sex, male (vs. female)	1.19 (1.18, 1.20)	<0.001
Having a job (vs. unemployment)	0.95 (0.94, 0.96)	<0.001
Household income level		
Q1 (Lowest) (vs. medical aid program)	0.88 (0.87, 0.90)	<0.001
Q2 (vs. medical aid program)	0.87 (0.86, 0.88)	<0.001
Q3 (vs. medical aid program)	0.84 (0.83, 0.85)	<0.001
Q4 (Highest) (vs. medical aid program)	0.81 (0.80, 0.82)	<0.001
Unknown (vs. medical aid program)	0.90 (0.87, 0.93)	<0.001
Residence		
Rural area (vs. Urban area)	1.01 (1.00, 1.02)	0.002
Unknown (vs. Urban area)	1.15 (1.11, 1.20)	<0.001
CCI, point	1.08 (1.08, 1.09)	<0.001
Admitting department		
IM (vs. non-IM)	1.45 (1.44, 1.46)	<0.001
Hospital admission through ER	1.44 (1.42, 1.45)	<0.001
Type of hospital		
General hospital (vs. tertiary general hospital)	1.21 (1.20, 1.22)	<0.001
Other hospital (vs. tertiary general hospital)	1.92 (1.87, 1.97)	<0.001
Surgery associated hospital admission	0.77 (0.77, 0.78)	<0.001
Mechanical ventilator support	3.37 (3.34, 3.40)	<0.001
ECMO support	2.00 (1.94, 2.06)	<0.001
CRRT use	2.46 (2.43, 2.49)	<0.001
Year of admission		
2011 (vs. 2010)	1.05 (1.03, 1.08)	<0.001
2012 (vs. 2010)	1.03 (1.01, 1.06)	<0.001
2013 (vs. 2010)	1.09 (1.06, 1.12)	<0.00
2014 (vs. 2010)	1.04 (1.01, 1.06)	<0.001
2015 (vs. 2010)	0.98 (0.95, 1.01)	0.125
2016 (vs. 2010)	0.98 (0.96, 0.99)	0.001
2017 (vs. 2010)	0.92 (0.91, 0.95)	<0.001
2018 (vs. 2010)	0.94 (0.91, 0.94)	<0.001
2019 (vs. 2010)	0.91 (0.90, 0.93)	<0.001
Main diagnosis		
Certain infectious and parasitic diseases	1	
Neoplasms	1.22 (1.20, 1.25)	<0.001
Diseases of the blood and blood-forming organs and certain disorders involving the immune mechanism	1.09 (1.03, 1.14)	0.003
Endocrine, nutritional and metabolic diseases	0.61 (0.59, 0.63)	<0.001
Mental, Behavioral and Neurodevelopmental disorders	0.39 (0.37, 0.42)	<0.001
Diseases of the nervous system	0.71 (0.69, 0.73)	<0.001
Diseases of the eye and adnexa	0.32 (0.25, 0.42)	<0.001
Diseases of the circulatory system	0.50 (0.50, 0.51)	<0.001
Diseases of the respiratory system	0.85 (0.84, 0.87)	<0.001
Diseases of the digestive system	0.71 (0.69, 0.72)	<0.001
Diseases of the skin and subcutaneous tissue	1.15 (1.07, 1.23)	<0.001
Diseases of the musculoskeletal system and connective tissue	0.44 (0.42, 0.45)	<0.001
Diseases of the genitourinary system	0.62 (0.61, 0.64)	<0.001
Certain conditions originating in the perinatal period	0.44 (0.33, 0.57)	<0.001
Congenital malformations, deformations and chromosomal abnormalities	0.24 (0.21, 0.27)	<0.001
Symptoms, signs and abnormal clinical and laboratory findings, not elsewhere classified	0.77 (0.75, 0.79)	<0.001
Injury, poisoning and certain other consequences of external causes	0.59 (0.57, 0.60)	<0.001
Codes for special purposes	1.11 (0.64, 1.91)	0.709
External causes of morbidity, factors influencing health status and contact with health services	1.08 (1.04, 1.13)	<0.001

ICU, intensive care unit; aHR, adjusted hazard ratio; CI, confidence interval; CCI, Charlson comorbidity index; IM, internal medicine; ER, emergency room; ECMO, extracorporeal membrane oxygenation; CRRT, continuous renal replacement therapy.

## Data Availability

Data will be available upon reasonable request to corresponding author.

## Supplementary Materials

The following supporting information can be downloaded at: https://www.mdpi.com/article/10.3390/ijerph20010081/s1, Table S1: The ICD-10 codes used to compute the Charlson comorbidity index, Table S2: 18 common specific diseases in the main diagnoses at ICU admission.
