# Supplementary material for: Epidemiologic Study of Intensive Care Unit Admission in South Korea: A Nationwide Population-Based Cohort Study from 2010 to 2019
Source: Int J Environ Res Public Health. 2022 Dec 21;20(1):81. doi: 10.3390/ijerph20010081 (PMC9819529; doi:10.3390/ijerph20010081)
Supplement: Supplementary file 1 [file ijerph-20-00081-s001.zip › Table S2.pdf]

Table S2. 18 common specific diseases in the main diagnoses at ICU admission

Main diagnosis	Number (%)
Pneumonia	140,511 (4.0)
Cerebral infarction	100,188 (2.8)
Unstable angina	96,868 (2.8)
Traumatic subdural hemorrhage	84,178 (2.4)
Acute myocardial infarction	81,691 (2.3)
Sepsis	65,388 (1.9)
Cerebral aneurysm, nonruptured	59,801 (1.7)
Chronic kidney disease, stage 5	47,221 (1.3)
Pneumonitis due to inhalation of food and vomit	44,816 (1.3)
Liver cell carcinoma	42,388 (1.2)
Angina pectoris	41,947 (1.2)
Nontraumatic intracerebral hemorrhage	37,569 (1.1)
Non-ST elevation myocardial infarction	36,034 (1.0)
Nontraumatic intracerebral hemorrhage	32,545 (0.9)
Heart failure	31,173 (0.9)
Acute kidney failure	26,711 (0.8)
Malignant neoplasm of bronchus or lung	26,616 (0.8)
Nontraumatic subarachnoid hemorrhage	24,906 (0.7)

ICU, intensive care unit
